# Effect of placenta location detected by ultrasound on the severity of placenta accreta spectrum in patients with placenta previa and placenta accreta spectrum

**DOI:** 10.1186/s12884-023-05736-w

**Published:** 2023-06-01

**Authors:** Hong Liu, Baolian Zhang, Wenli Wang, Haiyan Li, Xianghua Huang, Jia Wang, Jing Han, He Zhu

**Affiliations:** 1 Department of Physical Examination Center, The Second Hospital of Hebei Medical University, Shijiazhuang, Hebei China; 2 Department of Gynecology and Obstetrics, The Second Hospital of Hebei Medical University, Shijiazhuang, Hebei China; 3 Department of Ultrasound in Obstetrics and Gynecology, The Second Hospital of Hebei Medical University, Shijiazhuang, Hebei China; 4 Department of Quality Control, The Second Hospital of Hebei Medical University, Shijiazhuang, Hebei China

**Keywords:** Anterior placenta, Non-central placenta, Placenta accreta spectrum, Placental location, Placenta previa

## Abstract

**Background:**

To evaluate the effect of placental location on the severity of placenta accreta spectrum (PAS).

**Methods:**

We analyzed 390 patients with placenta previa combined with placenta accreta spectrum who underwent cesarean section between January 1, 2014 and December 30, 2020 in the electronic case database of the Second Hospital of Hebei Medical University. According to the position of the placenta, 390 placentas were divided into the posterior group (*n* = 89), the anterior group (*n* = 60) and the non-central group (*n* = 241).

**Results:**

The history of cesarean delivery rates in the anterior group (91.67%) and the non-central group (85.71%) were statistically different from the posterior group (63.74%)(*P* < 0.001). Univariate logistic regression results showed that employment, urban living, gestational age, complete placenta previa, fetal presentation shoulder, gravidity, cesarean section and vaginal delivery were all predictors for the severity of placenta accreta (*P* < 0.05). The anterior group (*P* = 0.001, OR = 4.13, 95%CI: 1.84–9.24) and the non-central group (*P* = 0.001, OR = 2.90, 95%CI: 1.55–5.45) had a higher incidence of invasive accreta placentation than the posterior group, and were independent risk factors for invasive accreta placentation.

**Conclusion:**

Compared with posterior placenta, anterior and non-central placenta are independent risk factors for invasive PAS in patients with placenta previa, during which we should be more cautious in treatment.

## Background

Placenta accreta spectrum (PAS) is a group of diseases in which placental tissue invades the myometrium. It is one of the most critical and severe obstetrics diseases, resulting in postpartum bleeding, secondary infection, multiple organ failure, perinatal hysterectomy, pelvic organ damage and other complications, even maternal and newborn deaths [1]. The incidence of PAS is increasing year by year [2]. A recent systematic review and meta-analysis of 23 cohort studies involving 350,939 women in mainland China found that the incidence of PAS increased from 0.03% in 1970–1979 to 0.48% in 2010–2016 [3]. PAS has many risk factors. The incidence of PAS increases with the increase of cesarean section history [4]. In addition, studies have found that other risk factors for placenta accreta include uterine surgery, in vitro fertilization, multiple births, and maternal age [5].

A unified appropriate diagnostic criterion for PAS is an important prerequisite for standardized clinical diagnosis and treatment and follow-up research. According to the definition, pathological diagnosis is the most objective and accurate, and the pathological grading criteria of PAS are constantly updated and verified [6, 7]. It is proposed that cases with hysterectomy and placenta in situ retention should be carefully examined and analyzed according to gross or microscopic manifestations. However, with the continuous improvement of clinical management of PAS, the number of hysterectomy is relatively reduced. In 2019, the relevant guidelines [8] were issued by the new International Federation of Gynecology and Obstetrics (FIGO) to define the intraoperative clinical grading criteria. At present, both intraoperative diagnosis and pathological diagnosis can be used as the diagnostic criteria for PAS. Invasive PAS refers to cases graded as grade 2 or higher, and non-invasive refers to cases graded as grade 1.

In recent years, abnormal placentation, especially placenta previa combined with PAS has become a hot research topic in obstetrics field [9]. There are few studies on the effect of placenta location on the severity of PAS in the patients with placenta previa. Therefore in this study, we aimed to evaluate the effect of placental location on the severity of PAS in the patients with placenta previa and PAS.

## Methods

We retrospectively analyzed 390 patients with placenta previa combined with placenta accreta who underwent cesarean section in the obstetrics department between January 1, 2014 and December 30, 2020 in the electronic case database of the Second Hospital of Hebei Medical University. Exclusion criteria were patients (1) with pregnancy complicates such as diabetes and hypertension; (2) with gestational age < 28 weeks and > 42 weeks; (3) with serious medical and surgical complications, such as malignant tumor; (4) with data loss. This study was approved by the Ethics Committee of the Second Hospital of Hebei Medical University (No. 2021–2178), and informed consent was obtained from each participant.

PAS was confirmed by intraoperative diagnosis or pathological diagnosis in all cases in this study. According to the FIGO guidelines, placenta accreta, placenta increta and placenta percreta are classified. Placenta increta and placenta percreta are collectively referred to as invasive accreta placentation according to their histological and clinical definitions [8]. Therefore the PAS was divided into two groups: adherent accreta placentation and invasive accreta placentation.

Clinical diagnostic criteria for adherent accreta placentation: Visually, there is no obvious distension of the uterus on the placental bed (placental bulge), no placental tissue invading the surface of the uterus, and no or minimal neovascularity. Manual removal of the placenta can cause heavy bleeding from the placenta accreta site requiring mechanical or surgical procedures. Clinical diagnostic criteria for invasive accreta placentation: Macroscopic abnormalities of the placenta bed are observed including bluish/purple colouring and distension (placental bulge). Gentle cord traction results in the uterus being pulled inwards without separation of the placenta (the dimple sign) [8].

All patients underwent two-dimensional ultrasound and color Doppler ultrasound examination before delivery. In present study, Ultrasound was done by ultrasound system, using the curvilinear transducer. The lower uterine segment is evaluated using the highest-frequency transducer that can produce an adequate image. Transabdominal ultrasound is performed with the patient's bladder full. The patients were positioned in a supine state on a chair, and the probe was applied with couplant before being placed on the patient's abdomen for exploration. Multi-section plain scanning of longitudinal and transverse sections of the abdomen was conducted using the GEVOLUSONE10 two-dimensional ultrasound to observe the internal echo of the placenta, the relationship between the placenta and the uterine muscle wall, the boundary state of the uterine myometrium, the relationship between the uterus and the bladder, and the smoothness of the bladder wall. Following this, the GEVOLUSONE10 color Doppler ultrasound was used to observe the blood flow within the placenta, between the uterine wall of the placenta, and between the uterine wall and the bladder, with key exploration parameters being recorded.

Placental location diagnosis: According to the position of the placenta examined by ultrasound in the week before surgery, 390 placentas were divided into three groups: the posterior group (*n* = 89), the anterior group (*n* = 60) and the non-central group (*n* = 241). Anterior wall placenta: More than 50% of the placental tissue is attached to the anterior wall. Posterior wall placenta: More than 50% of the placental tissue is attached to the posterior wall. Non-central placenta: cases other than anterior and posterior placenta. The risk of complications with the placenta on the posterior wall is lower than elsewhere, so the posterior wall group was selected as the control group [10].

### Statistical analysis

The statistical software Empower Stats (http://www.empowerstats.com, X&Y Solutions, Inc., Boston, MA) was used for statistical analysis. Continuous variables were expressed as mean ± standard deviation (SD), and differences between groups were compared by ANOVA. Categorical variables were represented by number (percentage), and differences between groups were compared by χ^2^ test. The p-value is obtained by Kruskal Wallis rank sum test for variables of skewed distribution. If the counting variable has a theoretical number < 10, the p-value is obtained by Fisher's exact probability test. Indicators and effect values were expressed with 95% confidence intervals. Univariate and multivariate logistic regression were used to explore the effect of placenta position on the degree of placenta accreta. In the basic model or the complete model, the covariates that had an impact on > 10% of the regression coefficient of X or regression coefficient *P* < 0.1 of Y were identified as confounders and adjusted in the multiple regression model. Two-tailed probability value of *P* < 0.05 was considered as statistically significant.

## Results

### Baseline data

As shown in Table 1, the factors affecting placenta attachment position include gestational age, gravidity, parity, number of vaginal delivery, type of placenta previa and number of cesarean delivery among patients with placenta previa combined with PAS. The results showed that the history of cesarean delivery rates in the posterior, anterior and non-central groups were 63.74%, 91.67% and 85.71%, respectively, and the anterior and the non-central groups are statistically different from the posterior group respectively (*P* < 0.001). Age, BMI, region, occupation, bleeding during pregnancy, emergency, multiple births, and fetal position had no effect on placenta position.

**Table 1:** Baseline data in three groups

	Posterior group (*n* = 89)	Anterior group (*n* = 60)	Non-central group (*n* = 241)	*P* value
Age(years)	31.02 ± 4.93	31.02 ± 4.93	31.27 ± 4.62	0.889
Gestational age(days)	252.63 ± 15.45	249.32 ± 12.62	247.05 ± 15.15	0.010
BMI	28.49 ± 4.08	27.90 ± 3.57	28.11 ± 3.82	0.619
Region, N (%)				0.418
Rural	60(67.42)	45(75.00)	158(65.56)	
Urban	29(32.58)	15(25.00)	83(34.44)	
Occupation, N (%)	30(33.71)	19(31.67)	77(31.95)	0.949
Gravidity, N (%)				0.002
1	10(11.24)	2(3.33)	7(2.90)	
2–3	45(50.56)	37(61.67)	108(44.81)	
> 3	34(38.20)	21(35.00)	126(52.28)	
Parity, N (%)				0.012
0	13(14.61)	4(6.67)	15(6.22)	
1	61(68.54)	44(73.33)	151(62.66)	
≥ 2	15(16.85)	12(20.00)	75(31.12)	
Uterine surgery, N (%)				0.013
0	35(39.33)	30(60.00)	69(28.63)	
1	29(32.58)	17(28.33)	79(32.78)	
> 1	25(28.09)	13(21.67)	93(38.59)	
Number of vaginal delivery, N (%)	23(25.84)	2(3.33)	34(14.11)	< 0.001
Hemorrhage during pregnancy, N (%)	51(57.30)	37(61.67)	163(67.63)	0.197
The type of placenta previa, N (%)				< 0.001
Marginal	26(29.21)	16(26.67)	0(0.00)	
Partial	13(14.61)	3(5.00)	5(2.07)	
Complete	50(56.18)	41(68.33)	236(97.93)	
Emergency surgery	21(23.60)	14(23.33)	49(20.33)	0.808
History of cesarean delivery, N (%)	57(64.04)	55(91.67)	208(86.31)	< 0.001
Fetal presentation				0.841
Cephalic	72(80.90)	51(85.00)	193(80.08)	
Breech	10(11.24)	4(6.67)	29(12.03)	
Shoulder	7(7.87)	5(8.33)	19(7.88)	
Grade of placenta accreta spectrum				< 0.001
Adherent	57(64.04)	15(25.00)	60(24.90)	
Invasive	32(35.96)	45(75.00)	181(75.10)	

According to the severity of placenta accreta, patients were divided into two groups: adherent accreta placentation group (*n* = 132) and invasive accreta placentation group (*n* = 258). As shown in Table 2**,** the gestational age, region, occupation, gravidity, parity, number of vaginal delivery, the type of placenta previa, history of cesarean delivery, fetal presentation, and placental location were significantly different in adherent accreta placentation and Invasive accreta placentation groups (*P* < 0.05).

**Table 2:** Comparison of basic data between two groups of people with different degree of PAS

	Adherent accreta placentation (*n* = 132)	Invasive accreta placentation (*n* = 258)	*P* value
Age(years)	31.05 ± 4.34	31.24 ± 4.72	0.704
Gestational age(days)	251.21 ± 15.99	247.37 ± 14.33	0.017
BMI	28.42 ± 4.00	28.03 ± 3.76	0.343
Region, N (%)			0.006
Rural	77(58.33)	166(72.09)	
Urban	55(41.67)	72(27.91)	
Occupation, N (%)			0.032
No	80(60.61)	187(71.65)	
Yes	52(39.39)	72(27.91)	
Gravidity, N (%)			< 0.001
1	14(10.61)	5(1.94)	
2- 3	62(46.97)	128(49.61)	
> 3	56(42.42)	125(48.45)	
Parity, N (%)			< 0.001
0	21(15.91)	11(4.26)	
1	87(65.91)	169(65.50)	
≥ 2	24(18.18)	78(30.23)	
Number of vaginal delivery, N (%)			< 0.001
0	100(75.76)	231(89.53)	
1	32(24.24)	27(10.47)	
Hemorrhage during pregnancy, N (%)			0.120
No	54(40.91)	85(32.95)	
Yes	78(59.09)	173(67.05)	
The type of placenta previa, N (%)			< 0.001
Marginal	27(20.45)	15(5.81)	
Partial	15(11.36)	6(2.33)	
Complete	90(68.18)	237(91.86)	
Emergency surgery	23(17.42)	61(23.64)	0.157
History of cesarean delivery, N (%)	85(64.39)	235(91.09)	< 0.001
Uterine surgeries			0.355
0	51(38.64)	83(32.17)	
1	37(28.03)	88(34.11)	
≥ 2	44(33.33)	87(33.72)	
Fetal presentation			0.030
Cephalic	103(78.03)	213(82.56)	
Breech	12(9.09)	31(12.02)	
Shoulder	17(12.88)	14(5.43)	
Placental location			< 0.001
Posterior	57(43.18)	32(12.40)	
Anterior	15(11.36)	45(17.44)	
Non-central	60(45.45)	181(70.16)	

The factors affecting the placenta attachment location in Table 1 and factors having an impact on the severity of placenta accreta indicated by previous studies were all subject to single-factor logistic regression for the degree of placenta accreta, and the positive results were shown in Table 3. Univariate logistic regression analysis was performed on the severity of PAS based on various influencing factors. The results showed that employment, urban living, gestational age, complete placenta previa, fetal presentation shoulder, gravidity, cesarean section and vaginal delivery were all predictors for the severity of placenta accreta (*P* < 0.05).

**Table 3:** Factors influencing the severity of placenta implantation in patients with placenta previa and PAS

	OR	95% confidence interval of Exp (β)		*P* value
		Lower limit	Upper limit	
In employment	0.62	0.40	0.96	0.033
Urban	0.54	0.35	0.84	0.0064
Gestational age	0.98	0.97	1.00	0.0176
Complete placenta previa	4.74	2.41	9.32	< 0.0001
Fetal presentation shoulder	0.40	0.19	0.84	0.0155
Gravidity 2- 3	5.78	1.99	16.77	0.0012
Gravidity > 3	6.25	2.15	18.20	0.0008
Cesarean section	5.65	3.24	2.46	< 0.0001
Vaginal delivery	0.37	0.21	0.64	0.0005
Uerine surgery 1	1.46	0.87	2.46	0.1517
Uerine surgery ≥ 2	1.21	0.73	2.01	0.4481

*OR* Odd ratio

**Fig. 1:**
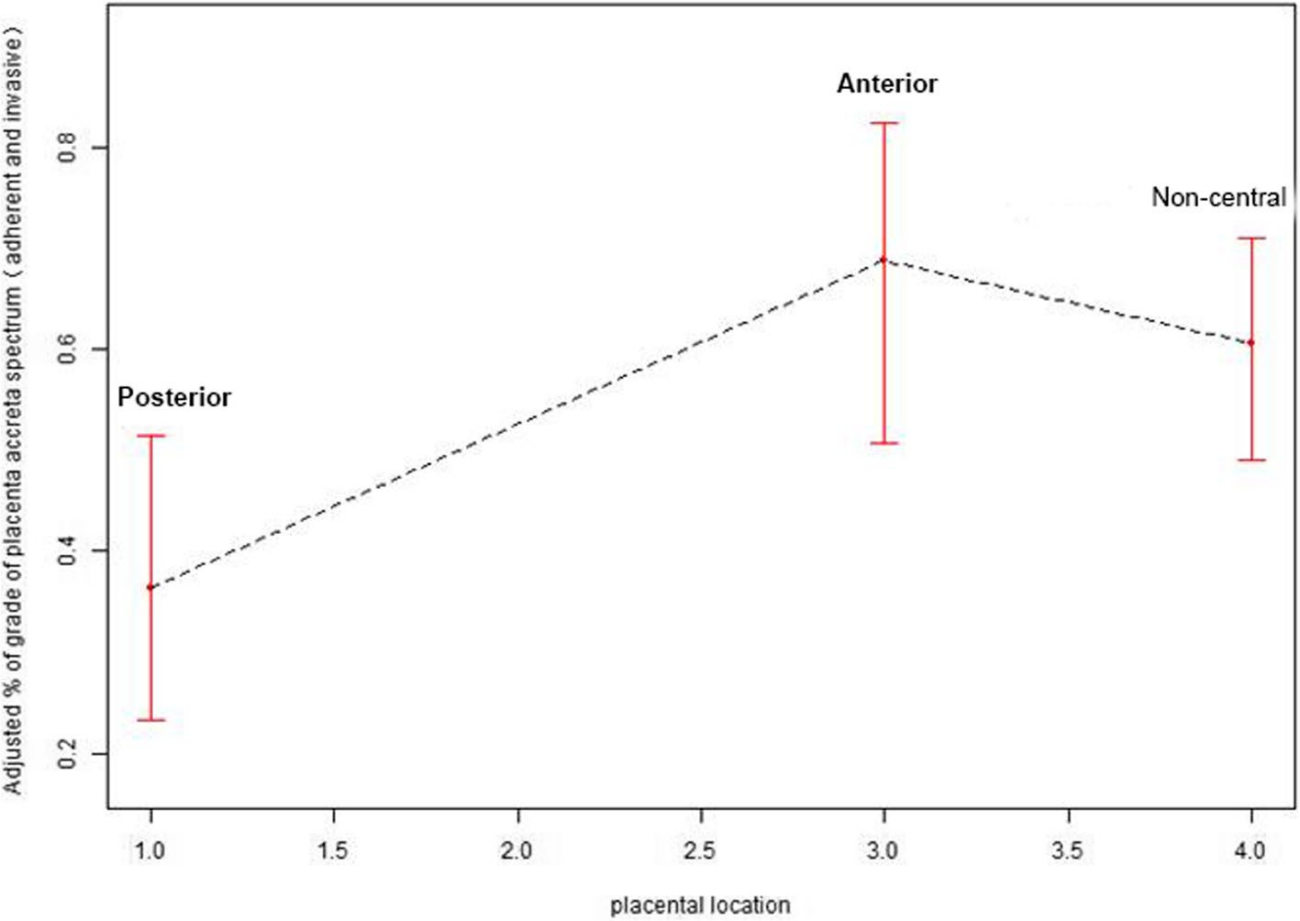
The fitting curve of placenta position and the severity of PAS in patients with placenta previa and PAS. The X-axis represents placental location. 1 is the posterior group, 3 is the anterior group, and 4 is the non-central 4 group. The Y-axis represents the incidence and 95% confidence interval of invasive placental implantation

As shown in Table 4, multiple logistic regression analysis was performed to analyze the influence of placental position on the severity of placenta accreta. When covariates were introduced into the base model or in the complete model, adjust I removed the covariates when their impact on the regression coefficient of X was > 10%. Adjust II eliminated the covariates, when their impact on the regression coefficient of X > 10% or the regression coefficient of Y *P* value < 0.1. The results showed that the anterior group (*P* = 0.001, OR = 4.13, 95%CI: 1.84–9.24) and the non-central group (*P* = 0.001, OR = 2.90, 95%CI: 1.55–5.45) had a higher incidence of invasive accreta placentation than the posterior group, and were independent risk factors for invasive accreta placentation. The curve fitting between placenta position and the severity of PAS was carried out, as shown in Fig. 1. It can be seen that placenta located on the anterior wall has a greater impact on the severity of PAS.

**Table 4:** Multivariate logistic regression analysis of the effect of placenta position on the severity of PAS in patients with placenta previa and PAS

Placental location	Posterior	Anterior		Non-central	
		*P*	OR(95%CI)	*P*	OR(95%CI)
Non-adjusted model	-	< 0.001	5.34(2.58,11.06)	< 0.001	5.53(3.19,9.06)
Model I	-	< 0.001	4.14(1.88,9.09)	0.001	2.88(1.57,5.27)
Model II	-	0.001	4.13(1.84,9.24)	0.001	2.90(1.55,5.45)

Non-adjusted model adjust for: None

Model I adjust for: type of placenta previa; cesarean section

Model I II adjust for: job; region; gestational age; type of placenta previa; fetal presentation; parity; cesarean section; number of vaginal delivery; gravidity; uterine surgery

Comparison of maternal and infant outcomes among the three groups was shown in Table 5. There were significant differences in operation time, intraoperative blood loss, blood transfusion, fetal weight and postoperative hospital stays among the three groups (*P*<0.05).

**Table 5:** Maternal and infant outcomes in 3 groups

	Posterior group (*n* = 89)	Anterior group (*n* = 60)	Non-central group (*n* = 241)	*P* value
Operation time(min)	100.0(80.0,135.0)	110.0(90.0,142.5)	120.0(95.0,157.0)	0.004
Intraoperative blood loss (ml)	800(600,1200)	1500(1000,2300)	1500(1000,2800)	< 0.001
Fetal weight(g)	2756.40 ± 545.43	2613.17 ± 493.18	2562.33 ± 552.22	0.016
Postoperative hospital stays(day)	5.00(5.00,6.00)	6.00(5.00,7.25)	6.00(5.00,7.00)	0.031
Hysterectomy, N (%)	2(2.25)	3(5.00)	22(9.13)	0.075
Transfusion, N (%)	50(56.18)	53(88.33)	215(89.21)	< 0.001
NICU, N (%)	43(48.31)	32(53.33)	141(58.51)	0.240
ICU, N (%)	3(3.37)	4(6.67)	19(7.88)	0.345
Complication, N (%)	10(11.36)	12(20.00)	54(22.41)	0.082

*NICU* Neonatal Intensive Care Unit, *ICU* Intensive care unit

Univariate regression analysis of maternal and infant outcomes between the anterior group and non-central group was shown in Table 6, and it was found that the intraoperative blood loss, transfusion and postoperative hospital stay in the anterior group and the non-central group were higher than the posterior group (*P* < 0.05), fetal weight in the non-central group was lower than that in the posterior group (β = -194.1 95%CI: -325.9,-62.2, *P* = 0.004), and maternal complications in the non-central group was higher than the posterior group (OR = 2.3, 95%CI:1.1,4.6, *P* = 0.028).

**Table 6:** Univariate analysis of maternal and infant outcomes

	Posterior group	Anterior group		Non-central group	
	(*n*=89)	(*n*=60)		(*n*=241)	
		OR(95% confidence interval)	*P* value	OR (95% confidence interval )	*P* value
Hysterectomy	1	2.3(0.4,14.1)	0.372	4.4(1.0,19.0)	0.049
Operation time	0	1.3(-17.8,20.2)	0.893	11.3(-2.8,25.3)	0.117
Intraoperative blood loss	0	735.3(246.9,1224.1)	0.003	1078(715.5,1441.2)	<0.001
Transfusion	1	5.9(2.4,14.4)	<0.001	6.4(3.6,11.6)	<0.0001
Fetal weight	0	-143.2(-320.7,34.2)	0.114	-194.1(-325.9,-62.2)	0.004
NICU	1	1.2(0.6,2.4)	0.548	1.5(0.9,2.5)	0.099
ICU	1	2.0(0.4,9.5)	0.36	2.5(0.7,8.5)	0.157
Postoperative hospital stays	0	0.9(0.2,1.6)	0.014	0.7(0.2,1.2)	0.011
Complication	1	1.9(0.8,4.9)	0.152	2.3(1.1,4.6)	0.028

*NICU* Neonatal Intensive Care Unit, *ICU* Intensive care unit

### Discussion

In this study, we found that placenta placement in the anterior and non-central wall has a greater impact on the severity of PAS than the posterior wall, after adjusted for confounding factors including region, parity, cesarean section, and placenta previa type. The risk of invasive accreta placentation for anterior placenta was 3.13 times higher than posterior placenta, and the risk of invasive accreta placentation for non-central placenta was 1.90 times higher than posterior placenta. Severe PAS indicated an increased risk of adverse maternal and infant outcomes including massive bleeding and hysterectomy [11, 12].

Cases of placenta previa with PAS were selected in this study. In the anterior group, 91.67% had a history of cesarean section. When the placenta is located on the anterior wall and in a low position, it is likely to cover the scar of the uterus. Study found that in the incision healing process of patients with cesarean section, more than 1/2 had the wedge-shaped healing defects [13]. The endometrium is damaged, and growed poor at the incision, and the muscularis is weak after cesarean section. Once the villi are implanted here, the bottom decidua is poorly formed, and trophoblast cells can directly invade the myometrium. The villi adhered to the myometrium, implanted and even penetrated the uterine wall [14]. The invasive PAS may occur as a result of partial or complete rupture of the uterine scar, resulting in deeper infiltration of villous trophoblast cells [15]. Therefore cesarean sections are also important factors in the severity of placental implantation. However, after adjusting the effect of cesarean section, the anterior placenta was still associated with invasive PAS in this study. This indicated that there were other reasons that affect the proliferation of placental villus and trophoblast cells, which have also been confirmed in a previous study [16], and the possible mechanism still needs to be further explored.

Jing L et al. found that there is an increased risk of adverse pregnancy outcomes and postpartum haemorrhagia in patients with placenta previa, when the placenta is located on the anterior wall [17]. Morgan E A et al. found that PAS with posterior placental location is associated with delayed diagnosis, surgical complications, assisted reproductive technology, and lower numbers of prior cesarean deliveries relative to anterior location [18]. Given that severe PAS indicated an increased risk of adverse maternal and infant outcomes [11, 12], these findings are similar to ours.

In addition, different parts of the uterus have different shapes and blood supply. The anatomy structure of other parts of the uterus is not as flat as the anterior and posterior walls of the uterus, which is not conducive to placenta attachment [19]. In order to absorb more nutrients to supply the fetus, the placenta will penetrate further into the myometrium. These may be the reasons why the non-central group had more severe PAS. In addition, complete placenta previa was observed in 97.55% of the non-central group. The whole placental tissue of complete placenta previa completely covered the entire os uteri, occupying a large area of the lower uterine segment, while the muscle layer of the lower uterine segment in pregnancy was thin [20]. On this basis, because the placenta has abundant blood transport, it is easy to have adverse pregnancy outcomes such as placenta implantation.

The history of cesarean section and placenta previa are independent risk factors for PAS, and the incidence of PAS will increase with the number of previous cesarean sections [21]. Meanwhile, the incidence of PAS in pregnant women with placenta previa increased compared with those without placenta previa [22]. This is also similar to the results of our study, indicating that cesarean section and placenta previa type were the major confounders of placenta position on the severity of placenta accreta. In addition, in this study, employment, urban living, gestational age, complete placenta previa, fetal presentation shoulder, gravidity, and vaginal delivery were all correlated with the severity of PAS, which is consistent with the results of previous studies [21, 23, 24]. The effect of low gestational age on the severity of PAS may be due to the early intervention of severe placental implantation. In addition, previous study found that in vitro fertilization, multiple pregnancy and maternal age are also risk factors for PAS [25], however this is inconsistent with this study, which may be related to the low number of positive cases in each group.

Clinical prediction model can be regarded as a quantitative tool for medical risk assessment and patient benefit assessment, as well as a means of precision medicine and individualized medicine, which can provide more intuitive and rational information for both doctors and patients. Evaluating patients with PAS is of paramount importance, but identifying the many risk factors involved can be challenging. Recent studies have developed clinical prediction models based on clinical data, highlighting the potential for timely risk stratification of the target population [26, 27]. A prospective cohort study constructed a model including four key variables (loss of clear zone, abnormal placental lacunae, placental bulge and bladder wall interruption) was shown to reliably predict presence and severity of PAS. This is the first time this has been demonstrated using the recently codified definitions of the US signs and disease definitions [28]. These findings highlight the need for large prospective studies aimed at exploring whether the clinical prediction model for PAS can improve the prenatal diagnostic accuracy and surgical outcome.

There were some limitations of this study. This study lacks complete, detailed and effective information on placenta location. Except for placenta previa, there is no official classification of placenta location. In our study, it might be inaccurate to divide the placenta into three positions based only on transabdominal ultrasound examination, without combining transvaginal ultrasound examination. In addition, there were no patients without PAS were included as a control group. In the future, we will further combine risk factors to establish a prenatal prediction model for the degree of placenta accreta, so as to improve the accuracy of prenatal diagnosis of placenta accreta.

## Conclusions

In conclusion, we found that the anterior placenta and the non-central placenta are independent risk factors for invasive PAS in patients with placenta previa, during which we should be more cautious in treatment. Prenatal diagnosis of placental implantation degree is a complex problem. Next, we will collect more data and create a clinical prediction model for prenatal diagnosis of placental implantation degree, providing a basis for clinical treatment.

## Data Availability

The datasets generated during and analyzed during the current study are not publicly available, but are available from the corresponding author on reasonable request.
